# Comprehensive sequence-to-function mapping of cofactor-dependent RNA catalysis in the *glmS* ribozyme

**DOI:** 10.1038/s41467-020-15540-1

**Published:** 2020-04-03

**Authors:** Johan O. L. Andreasson, Andrew Savinov, Steven M. Block, William J. Greenleaf

**Affiliations:** 1 Department of Genetics, Stanford University, Stanford, CA 94305 USA; 2 Department of Biochemistry, Stanford University, Stanford, CA 94305 USA; 3 Biophysics Program, Stanford University, Stanford, CA 94305 USA; 4 Department of Biology, Stanford University, Stanford, CA 94305 USA; 5 Department of Applied Physics, Stanford University, Stanford, CA 94305 USA; 6 Chan Zuckerberg Biohub, San Francisco, CA USA; 7 Present Address: Department of Genome Sciences, University of Washington, Seattle, WA 98195 USA

**Keywords:** RNA, High-throughput screening, Ribozymes

## Abstract

Massively parallel, quantitative measurements of biomolecular activity across sequence space can greatly expand our understanding of RNA sequence-function relationships. We report the development of an RNA-array assay to perform such measurements and its application to a model RNA: the core *glmS* ribozyme riboswitch, which performs a ligand-dependent self-cleavage reaction. We measure the cleavage rates for all possible single and double mutants of this ribozyme across a series of ligand concentrations, determining *k*_cat_ and *K*_M_ values for active variants. These systematic measurements suggest that evolutionary conservation in the consensus sequence is driven by maintenance of the cleavage rate. Analysis of double-mutant rates and associated mutational interactions produces a structural and functional mapping of the ribozyme sequence, revealing the catalytic consequences of specific tertiary interactions, and allowing us to infer structural rearrangements that permit certain sequence variants to maintain activity.

## Abstract (web summary)

RNA sequence-structure-function relationships can be studied via assays of variant activity. The authors developed a method to systematically determine ribozyme variant kinetics, using it to map double-mutant interactions and show that cleavage rate drives sequence conservation in the *glmS* ribozyme.

## Introduction

Across all three domains of life, evolution has generated a diverse set of functional RNAs known to play important roles in cellular homeostasis and gene regulation^1–6^. These biopolymers fold into modular, three-dimensional structures capable of binding specific ligands (aptamers) or catalyzing specific biochemical reactions (ribozymes). Some of these RNAs, called riboswitches, act as cellular biosensors, altering gene expression in response to levels of specific ligands or temperature conditions^7^. A deeper understanding of the relationships between the sequences, structures, and activities of naturally evolved RNA switches would greatly aid in determining design principles for synthetic biosensors. An engineered RNA biosensor might, for example, couple into a single functional RNA the ability to catalyze a desired chemical reaction in response to a specific environmental cue.

The self-cleaving *glmS* riboswitch supplies a naturally occurring example of just such a functional combination, and is therefore a good system for investigating the underlying sequence–structure–function relationship of such RNAs, and discovering design principles. Commonly found in the 5′ UTR of the mRNA coding for the enzyme glucosamine-6-phosphate synthase^8,9^, the *glmS* riboswitch binds the metabolite glucosamine 6-phosphate (GlcN6P)—the product of the synthase reaction—which then serves as a cofactor in performing self-cleavage^10–13^. RNA cleavage, in turn, targets the 3′ product fragment for subsequent degradation by RNase J1, preventing the message for the synthase enzyme from being translated^14^. This feedback mechanism helps to maintain appropriate cellular levels of GlcN6P, an essential precursor for bacterial cell-wall synthesis^15^.

The *glmS* ribozyme riboswitch has been characterized by crystallography^11,16,17^, but atomic-level structural information provides limited insight into the manner by which sequence changes might modulate either ligand binding or the catalysis of self-cleavage. Of course, nucleotides situated near binding pockets or active sites may be inferred to be important for function, but a predictive understanding of how sequence perturbations affect the distinct biophysical processes of ligand binding and catalysis, and which sequence changes introduce structural rearrangements that nevertheless maintain overall function, is much more challenging to obtain.

The advent of highly parallelized methods for systematically characterizing RNA function has provided a path forward^18–20^. These methods, which rely on high-throughput sequencing techniques, allow characterization of ribozyme function across a diverse sequence space, in some cases including the majority of possible single and double mutations. Single-nucleotide perturbations can be used to directly associate individual bases with functional consequences, while higher-order mutations can supply useful information about interactions among bases forming cooperative (and anti-cooperative) associations within the folded structure^18^. In principle, higher-order mutations may also reveal sequence variants that generate alternative folds. In addition, high-throughput measurements of sequence variant function facilitate a systematic comparison of mutational effects with biological sequence-conservation patterns, shedding light on selective pressures driving the evolution of functional RNAs. The most direct assessments of RNA sequence-function landscapes to date have been based on measurements of the fraction of ribozyme molecules cleaved at the reaction endpoint^20–23^. We have built upon this work by developing a chip-based assay capable of following the kinetics of the cleavage reaction in real time for every sequence variant produced.

**Fig. 1:**
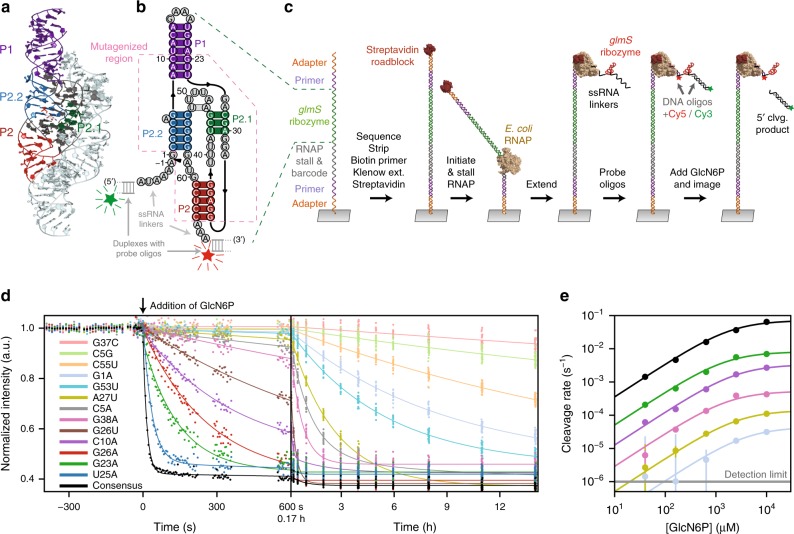
RNA array assay for ribozyme self-cleavage. **a** Crystal structure of the *T. tengcongensis glmS* ribozyme, PDB 2Z75, with duplex elements P1, P2, P2.1, and P2.2 indicated (core domain: dark colors; non-core domain, pale cyan). **b** Secondary structure of the 66-nt core construct studied; duplex color scheme same as in **a**. Flanking sequences and the region targeted for random mutagenesis by doped synthesis are also indicated. The sequence of the core construct is from ref. ^24^; the secondary structure is derived from crystallographic data (refs. ^11, 16^). **c** Schematic of sequential steps for the RNA array assay. Variants were transcribed in situ on a sequencing chip. Hybridized Cy3- and Cy5-dye labeled DNA oligomers scored the presence of the ribozyme. The Cy3 TIRF signal is lost upon cleavage, which releases the 5′ product. **d** Representative cleavage records for the consensus ribozyme and the 13 variants are indicated (color-coded), displayed on both short and long time scales (data points are median per-tile intensity values for the given variant). **e**, Representative Michaelis–Menten curve fits to cleavage rates, derived from RNA array data (as in **d**), as a function of GlcN6P concentration (error bars, std. err.). Same color code as in panel **d**. Source data are available in the Source Data file.

In general, the activity of a ligand-dependent ribozyme may be parameterized by (1) its catalytic rate, *k*_cat_, and (2) the associated Michaelis–Menten constant, *K*_M_. Here, we developed an in vitro assay to measure the activity of sequence variants of the consensus core *glmS* ribozyme^24^, a minimal model system, using an RNA array^25–27^ (Fig. 1a, b). This assay repurposes hardware originally developed for high-throughput sequencing to permit fluorescence-based measurements of endonucleolytic cleavage at selected ligand concentrations. Using the assay, we measure the raw cleavage rates, *k*_obs_, for ~24,000 *glmS* ribozyme sequence variants, including nearly all possible single and double mutants, as well as ~12,000 higher-order mutants. By fitting the observed reaction kinetics as a function of ligand concentration to the Michaelis–Menten model, we also determine the values of *k*_cat_ and apparent *K*_M_ for most functional variants. The values of *k*_obs_, *k*_cat_, and *K*_M_ for point mutants reveal the quantitative contributions of each residue to overall catalytic activity. We find that mutation frequency across bacterial *glmS* ribozyme sequences is strongly correlated with *k*_cat_, but not significantly correlated with *K*_M_, suggesting that the cleavage step, rather than ligand-sensitivity, largely drives the conservation of sequence. An analysis of pairwise mutational effects provides evidence for a significant number of functional interactions between residues and identified structural features crucial for catalysis, as well as suggesting a variety of structural rearrangements that enable the *glmS* ribozyme to maintain catalytic activity in the face of certain mutations. Overall, our approach complements both structural data and sequence-conservation analysis in formulating an improved understanding of the sequence-structure-function relationship for structured RNA.

## Results

### RNA array experiments probe effects of sequence on catalysis

We prepared a DNA library of transcription templates encoding sequence variants of the core *glmS* ribozyme using doped (error-prone) solid-phase synthesis and bottlenecking (Methods). As generated, the library included the consensus sequence along with all possible single mutants and essentially all (99.8% of possible) double mutants in a 54-nt portion of the core ribozyme, excluding the top half of the P1 hairpin (Fig. 1b, Supplementary Fig. 1). Ribozyme variant clusters were produced by in situ transcription from DNA clusters on Illumina sequencing chips, and self-cleavage kinetics were measured by monitoring the fluorescence decay of a Cy3-labeled DNA probe that was hybridized to the 5′-side of the cleavage dinucleotide. A second, Cy5-labeled DNA probe was hybridized to the 3′ side of the cleavage site to allow for verification of individual cluster positions, independent of the progress of the cleavage reaction (Fig. 1c) (Methods). To isolate the cleavage reaction from the ribozyme folding step^28^, we allowed >20 min for RNA folding to take place prior to the introduction of the ligand, GlcN6P, into the array. The assay allowed measurements of cleavage rates spanning five orders of magnitude, with variant *k*_obs_ values ranging from 1.5 × 10^−1^ s^−1^, a value close to the consensus-sequence rate at 10 mM GlcN6P (6.4 × 10^−2^ s^−1^), to the minimum discernible rate of ∼1 × 10^−6^ s^−1^ (Fig. 1d). Our results are in agreement with other determinations of the core ribozyme cleavage rate, including both bulk (∼6 × 10^−2^ s^−1^; Supplementary Fig. 1d) and single-molecule (∼3 × 10^−2^ s^−1^; ref. ^29^) measurements.

Across all sequence variants at 10 mM GlcN6P, 39% (72/183) of single mutants, 5% (604/12,063) of double mutants, and 0.8% (93/11,989) of higher-order mutants exhibited cleavage rates that were within an order of magnitude of the consensus ribozyme rate. No variant was found to cleave at a rate greater than 2.4 times that of the consensus form. A substantial proportion of variants were strongly detrimental to catalysis, with rates ≥ 10^4^-fold below the consensus value (≤6.4 × 10^−6^ s^−1^): these included 19% (35/183) of single mutants, 55% (6616/12,063) of double mutants, and 67% (8085/11,989) of higher-order mutants. We performed measurements at multiple concentrations of GlcN6P, which allowed us to determine values for *k*_cat_ and *K*_M_ (Fig. 1e) (Methods). Consistent with previous reports^12,28,30^, we found that the apparent *K*_M_ of the consensus ribozyme is ~2.0 mM (with 10 mM GlcN6P representing a saturating ligand condition). We successfully obtained values of *k*_cat_ for 83% (152/183) of single and 50% (6085/12,063) of double mutant variants, and apparent *K*_M_ values for 74% (135/183) of single and 33% (3996/12,063) of double mutant variants. Variants for which we were unable to derive kinetic parameters generally displayed extremely low cleavage rates at 10 mM GlcN6P (e.g., 55% of double mutants).

### Variations in self-cleavage rate drive sequence conservation

**Fig. 2:**
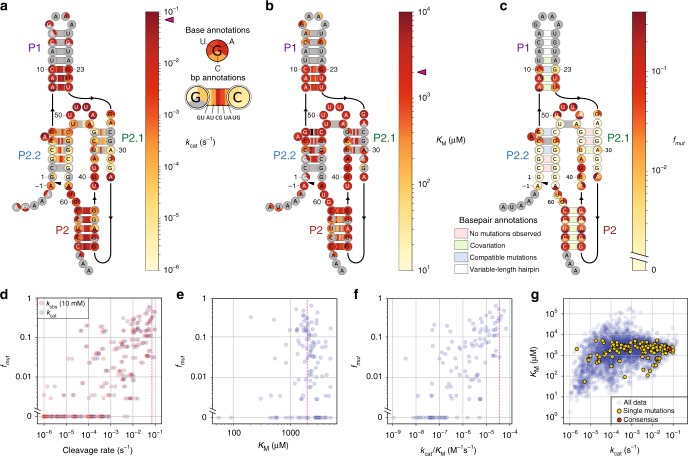
Mutation frequency is tightly correlated with effects on the cleavage rate. Structural heatmaps of *k*_cat_ (**a**) and apparent *K*_M_ (**b**) for all single mutants and basepaired-partner double mutants. Magenta arrowheads indicate consensus ribozyme values. **c** Structural heatmap of mutation frequency for point mutants, and previously reported basepair covariation, from ref. ^9^ (Methods). **d**–**f** Scatterplots of mutation frequency vs. cleavage rate (**d**), *K*_M_ (**e**), and *k*_cat_/*K*_M_ (**f**) for point mutants of the ribozyme core. Dotted magenta lines indicate consensus ribozyme values. **g** Scatterplot of *K*_M_ vs. *k*_cat_ for all variants for which both parameters were determined. Source data are available in the Source Data file.

To gain a better understanding of the functional underpinnings of ribozyme sequence conservation, we compared the results of our measurements for all single mutants and basepair double mutants with the variations found in an extensive set of biological *glmS* ribozyme sequences, based on a previous bioinformatic analysis^9^ (Fig. 2a–g) (Methods).

The pattern of ribozyme variant cleavage rates observed in this study closely mirrored the natural pattern of biological sequence conservation. Specifically, for single mutations of the consensus sequence, *k*_cat_ and *k*_obs_ (at saturating GlcN6P, 10 mM) were both strongly correlated with the mutation frequency, *f*_mut_ (for *k*_cat_, Spearman’s *ρ* = 0.739, *P* < 2 × 10^−25^ from two-tailed *t*-test; for *k*_obs_(10 mM), Spearman’s *ρ* = 0.796, *P* < 1 × 10^−36^ from two-tailed *t*-test) (Fig. 2a, c, d; Supplementary Fig. 2a, b). In fact, *k*_obs_ was strongly correlated with *f*_mut,_ across all measured ligand concentrations (e.g., for *k*_obs_ at 40 μM GlcN6P, Spearman’s *ρ* = 0.767, *P* < 2 × 10^−32^ from two-tailed *t*-test) (Supplementary Fig. 2c, Supplementary Table 1). On the other hand, the apparent *K*_M_, where measurable, varied little from the consensus-sequence value (2.0 mM) for single and basepair double mutants, and exhibited no significant correlation with *f*_mut_ (Spearman’s *ρ* = − 0.082, *P* = 0.369 from two-tailed *t*-test) (Fig. 2b, c, e). We estimated that 83% of measured *K*_M_ values for double mutants were within fivefold of the consensus value (Supplementary Fig. 3). As a consequence of the reduced variation in apparent *K*_M_, the catalytic efficiency of the ribozyme, expressed as *k*_cat_/*K*_M_, followed the same pattern as saturating cleavage rate, causing the efficiency to be strongly correlated with *f*_mut_ (Spearman’s *ρ* = 0.723, *P* < 4 × 10^−21^ from two-tailed *t*-test) (Fig. 2f). These findings for *k*_cat_ and *k*_cat_/*K*_M_ are consistent with the strong correlation between *f*_mut_ and *k*_obs_ at all GlcN6P concentrations, noted earlier. Across all measured single and double mutants, *k*_cat_ and apparent *K*_M_ are only weakly correlated (Spearman’s *ρ* = 0.272, *P* < 4 × 10^−77^ from two-tailed *t*-test) (Fig. 2g).

### Conservation does not always imply large catalytic effects

Although biological sequence conservation broadly correlated with cleavage rate and catalytic efficiency, we did observe some interesting exceptions to this trend. Nucleotide G23, situated near the base of P1, exhibited little sequence variation, yet G23 single mutants only reduced *k*_obs_(10 mM) by ∼11-fold, with equivalent results for *k*_cat_ (Fig. 2a, c, Supplementary Fig. 2b). The apparent *K*_M_ values for G23 mutants were consensus-like (Fig. 2b). The conservation pressure on G23 may be rationalized by considering its role in promoting full-length ribozyme folding at low Mg^2+^ concentrations, since G23 forms a base-triple interaction with a tetraloop of the folding-assisting auxiliary domain^11,31^. Because our experiments were performed on the core ribozyme at physiological Mg^2+^ concentrations, our measurements would not have been sensitive to such an effect. More generally, mutations that induced a mispairing at the base of P1 had little catalytic effect, despite covariation of the relevant basepairs (Fig. 2a, c). This finding is consistent with conservation of the P1 base being driven by ribozyme folding rate, with P1 acting to nucleate the folding of the P2.2–P2–P2.1 double pseudoknot^29^ (Supplementary Discussion).

### Insertions are less disruptive than deletions or mismatches

**Fig. 3:**
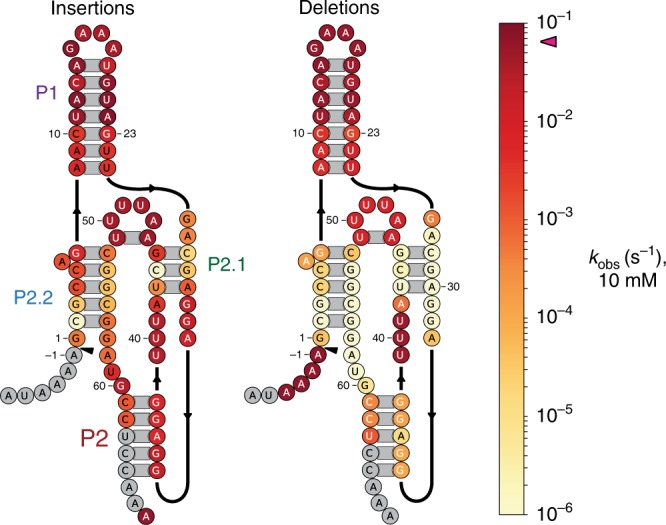
Catalytic consequences of insertions and deletions. Heatmap of the catalytic rates for double-incorporation insertion and point deletion mutants of the core *glmS* ribozyme. Source data are available in the Source Data file.

Owing to its construction based on solid-phase DNA synthesis, our library also included a nearly complete set of all possible point deletions and double-incorporation insertions, i.e., point insertions of a second copy of the expected base at a given position (Fig. 3). Point deletions were generally more detrimental than base-altering point mutations, but followed the same trend; within P2, deletions were poorly tolerated compared to base changes, a result likely explained by the increased difficulty for catalytically important bases in P2.2 or P2.1 to maintain base-pairing register (Fig. 3, Supplementary Fig. 2b). Double-incorporation insertions were better tolerated than either point deletions or base changes, but their effect on cleavage rate followed the same general pattern as that of base changes. We also analyzed the effects of higher-order mutants involving multiple deletions, or combinations of deletions and mismatches (Supplementary Fig. 4). Among other things, we found that up to eight deletions (mostly in the P1 stem) were tolerated, giving reasonable ribozyme activity. The existence of functional variants with so many nucleotides removed suggests that a truly minimal *glmS* ribozyme is even shorter than the consensus core construct established by earlier work and studied here (Supplementary Discussion, Supplementary Fig. 4).

### Double mutant cleavage rates reveal mutational interactions

**Fig. 4:**
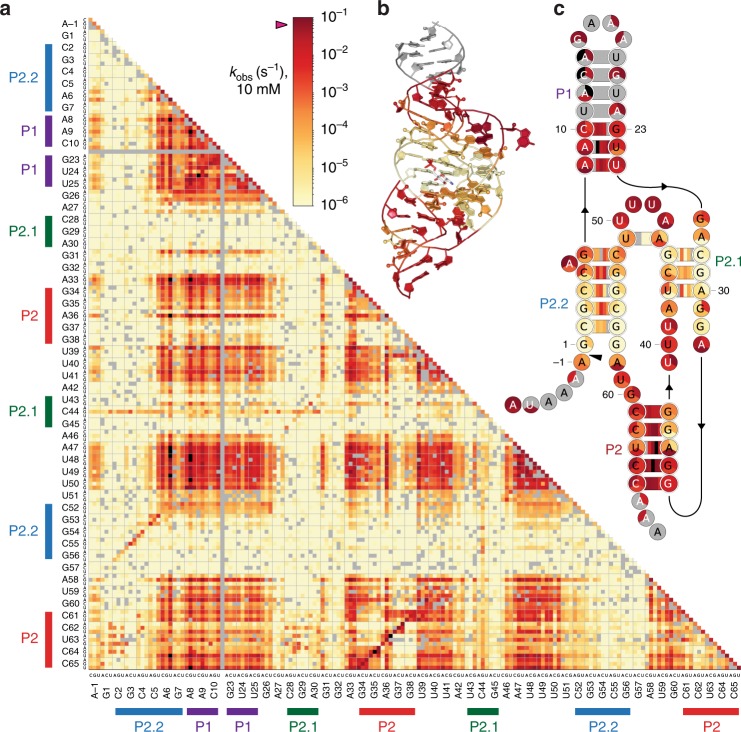
Effects of mutations on cleavage rate at 10 mM GlcN6P. **a** Heatmap of rates for all measured single and double mutants, with the first constituent point mutation indicated on the ordinate and the second on the abscissa. Sequence regions participating in duplex structures are labeled and color-coded as in Fig. 1. Heatmap of cleavage rates of all single mutants and basepaired-partner double mutants superimposed on the ribozyme tertiary (**b**) and secondary (**c**) structure, with secondary elements indicated in the same color scheme as **a**. The structure in **b** is based on PDB 2Z75. Source data are available in the Source Data file.

We quantified mutational interactions between all pairs of residues in the ribozyme through measurements of double-mutant rates. The heatmap of *k*_obs_(10 mM)(Fig. 4a) contains several immediately appreciable features. The hypotenuse of the plot represents cleavage rates for all point mutants; these are also plotted overlaid with the ribozyme’s tertiary (Fig. 4b) and secondary (Fig. 4c) structures for reference. Diagonal features oriented perpendicular to the hypotenuse reflect functional ribozyme variants with alternative basepairs, generated by double mutations within one of the duplex elements: P2.2, P1, P2.1, or P2 (Fig. 4a). Signatures of such alternative basepairing modes may also be found in the secondary structure heatmap (Fig. 4c). Rectangular “blocks” of double mutants enriched for self-cleavage activity, e.g., A47-U50 × A8-U25 (Fig. 4a), generally reflect combinations of single mutations that each fail to substantially affect self-cleavage activity in isolation. Residues exhibiting point mutations highly detrimental to catalysis were clustered around the active site in the crystal structure, whereas residues exhibiting point mutations with near-consensus rates generally lay more distal to the active site (Fig. 4b). For the cleavage dinucleotide A(–1)G1, we found that mutations in A(–1) were substantially better tolerated than mutations in G1 (Fig. 4c), consistent with prior reports^8,17^.

**Fig. 5:**
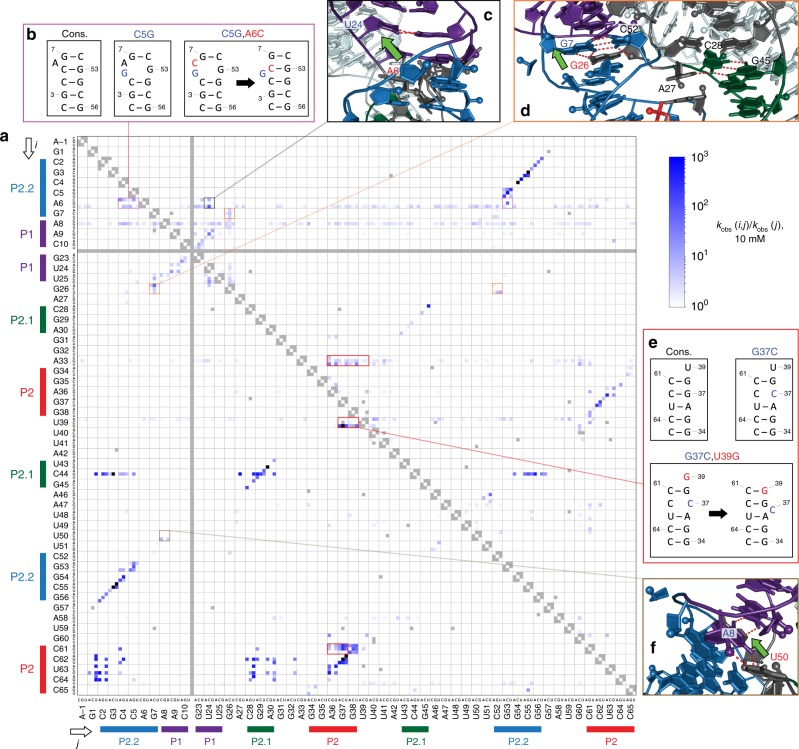
Double-mutant rescue interactions (for 10 mM GlcN6P). **a** Heatmap of double-mutant rescue, as defined in the main text. Sequence regions participating in duplex structures are labeled and color-coded, as in Fig. 4. **b**–**f** Examples of rescue interactions, illustrating different candidate mechanisms: secondary-structure rearrangements (**b, e**), tertiary contacts seen in crystal structures (**d, f**), and novel tertiary interactions (**c**). The crystal structure in **c**, **d**, and **f** is PDB 2Z75. Source data are available in the Source Data file.

If mutations acted independently, then a double mutant would be expected to exhibit a rate reduction equal to the product of the rate reductions of the two constituent point mutations. Therefore, double mutants with *k*_obs_(double)/*k*_obs_(single) > 1 (for a given constituent single mutant) were considered to display a “rescue” interaction. Accordingly, we constructed a heatmap of double-mutant rescue effects, with second-site mutations displayed on the vertical axis rescuing single mutations on the horizontal axis (Fig. 5a), which allowed rapid identification of mutational interactions in the dataset (Fig. 5b–f).

### Basepair mutants reveal structural and catalytic functions

As noted previously, diagonally oriented features of the heatmap (Fig. 4a) correspond to alternative basepairing double mutants within the duplex elements, P2.2, P1, P2.1, and P2. In a similar fashion, diagonal elements in the rescue map (Fig. 5a) highlight the rescue of mutations in consecutive nucleotides in these duplexes by compensatory mutations in their base-pairing partners. In fact, the secondary structure of the ribozyme can be inferred in its entirety simply from these diagonal features displayed in the rescue plot.

The patterns of double-mutant rescue effects and rates revealed in the heatmaps indicate a concurrence of our measurements with much of the available structural and biochemical evidence, and therefore help to illustrate the power of our technique. We first consider results for the P2.2 element: mutations here, and particularly in the three basepairs proximal to the cleavage site, were strongly detrimental to catalysis (Fig. 4c), but could be partially rescued (to <35-fold below consensus activity) by compensating mutations (Figs. 4a, 5a). Consistent with our findings, P2.2 was previously proposed to play a key role in positioning the cleavage site and cofactor^17^, and more recently, the folding of this element was demonstrated directly to control catalysis^29^. We also found that the bottommost basepair of P2.2 (C2•G56) resulted in low activity (>850-fold below consensus) when changed to any alternative basepair (Fig. 4a), even though the significant levels of double-mutant rescue (Fig. 5a) indicate that alternative basepairs at this position are superior to a mismatch. The identities of nucleotides at positions 2 and 56 would therefore seem to be important for catalysis, in addition to their structural roles in forming a duplex. This result is again consistent with structural data, which indicate that both these residues help form the active site, with C2 making H-bond contacts to the A(–1)G1 substrate^11^. Similarly, the limited ability to compensate for P2.1 mutations with base-pairing partner mutations (Fig. 4a) suggests that the nucleotides in P2.1 likely play direct roles in catalysis, in addition to serving other structural functions. This inference is fully consistent with the first-strand residues (28–30) of P2.1 helping to form the active site, and the second-strand residues (43–45) forming part of the cofactor binding pocket^11^. In contrast to the more nuanced results for P2.2 and P2.1, mutations in both P1 and P2 are rescued to consensus-like activity by compensatory base-pairing partner mutations (Fig. 4a, Fig. 5a), consistent with these elements playing purely structural roles—as might be anticipated from their placement in the crystal structure (Fig. 1a).

### Catalytic relevance of tertiary interactions

Two core-domain base triples have been previously identified from structural data, namely, the 26*7•52 triple and the 27*28•45 triple^11,16^, where “•” denotes a Watson–Crick basepair, and “*” indicates a non-Watson-Crick basepair. However, the significance of these structural contacts for catalytic activity had not been determined previously. Our data reveal prominent mutational interactions that can occur within 26*7•52, with G26 mutations rescuing second-site mutations in both members of the G7•C52 basepair (e.g., G26A rescues G7C by ~60-fold) (Fig. 5a, d), underscoring the importance of this tertiary interaction for catalytic function. Conversely, we did not observe rescue interactions within the 27*28•45 base triple. This is likely because C28 and G45 are components of the active site and the cofactor binding pocket respectively^11^. Thus, in addition to their structural role, the identities of residues at these positions are critical for binding and catalysis. We identified a further tertiary contact, which is also evident in published crystal structures^11,16,17^: residue 50 of the P2.1–P2.2 pseudoknot loop (residues 47–50) juts into the base of the P1 hairpin, positioning it to hydrogen-bond with residue 8 of the 8•25 basepair. Double-mutant rescue results indicate an interaction between residues 50 and 8, and specifically, an approximately sevenfold rescue of A8C by U50G (Fig. 5f). Thus, our measurements supply evidence that this additional tertiary interaction is important for catalysis.

### Mutational interactions suggest a novel tertiary interaction

We observed a single substantially detrimental point mutation in the base of the P1 hairpin: U24C, which reduces activity at saturating ligand by ~160-fold (Fig. 4c). We found that U24C was rescued by A6U, which improved activity 90-fold, to within half of the consensus value (Fig. 5a, c). Residue 6 is ~10 Å away from the 9•24 basepair in crystal structures^11,16,17^, too far for direct physical interaction, thereby raising the question of what mechanism might explain such long-range compensation. In the structure, the nucleobase of residue 6 is flipped out, away from the P2.2 and P1 helix interiors^11,16,17^; however, a structural rearrangement that flipped this base inward could plausibly bring it into interaction with the nucleobases of residues 9 and 24 (Fig. 5c). Thus, we conjecture that the A6U–U24C rescue interaction reflects a novel tertiary contact that enhances catalytic activity. Intriguingly, A6U also rescues U50C by sixfold, to within threefold of consensus-sequence activity. As noted, there is a catalytically important tertiary contact between residues 50 and 8 (Fig. 5f), and an unexpected contact between a flipped-in A6U and the 9•24 basepair might compensate for loss of the U50-A8 contact, helping to maintain self-cleavage activity.

### Interactions within P2.2 suggest alternative structures

Deletion of the bulged P2.2 nucleotide, A6, was substantially detrimental, whereas mismatches at this site had very little effect (Fig. 3, Supplementary Fig. 2b). The presence of a bulged nucleotide at this position is clearly important for catalysis, consistent with its ≥97% conservation across bacterial ribozyme sequences^9^. One function of this bulged nucleotide may be reflected by its ability to compensate for mutations elsewhere in P2.2: mutations in A6 rescued mutations in the adjacent C4, C5, and G53 residues, as exemplified by a 15-fold rescue of C5G by A6C (Fig. 5a). We interpret this rescue as arising from a secondary structural rearrangement in which C5G bulges out, thereby allowing A6C to pair with G53 (Fig. 5b). Similarly, A6C rescued C4A by ~24-fold (Fig. 5a), presumably by an analogous rearrangement, with C4A as the bulged nucleotide; and A6G rescued G53C by ~60-fold, in which case we infer C5 becomes the bulged residue. These results suggest that alternative P2.2 structures with bulges shifted towards the center of the duplex may have substantially improved cleavage activity compared with P2.2 duplexes carrying mismatches.

We note that the 5•53 basepair is not well-conserved, nor are C5 and G53 individually^9^ (Fig. 2c), yet mutations in either of these residues were substantially detrimental to catalysis, exhibiting an average ~10^3^-fold reduction in saturating cleavage rate across all single mutants (Fig. 4c). Of all point mutations to 5•53, only the wobble-pair-producing mutant C5U caused less than a 200-fold loss of activity. We hypothesize that nucleotides at positions 6, 5, 4, and 53 exhibit covariation due to mutational interactions between residue 6 and its neighbors, leading to the observed lack of correlation between activity and sequence conservation for the 5•53 basepair.

### P2 is subject to mutation-induced structural rearrangements

Similar to results for P2.2, we observed mutational interactions in P2 that appear to reflect its ability to rearrange structure while retaining function. In fact, P2 appeared to be particularly prone to such rearrangements, with numerous interactions observed. In one striking case, U39G rescued the inactive mutant G37C (*k*_obs_(10 mM) ~2 × 10^−6^ s^−1^) to within 30-fold of consensus activity (Fig. 5a, Fig. 4a). G37C would normally lead to a C–C mismatch within P2, but U39G presumably allows for a rearrangement where G37C bulges out, re-generating a version of P2 without internal mismatches (Fig. 5e).

Mutation U39G also rescued other changes in P2, in residues G37, G38, and C61 (Fig. 5a). Similarly, A33 exhibited strong mutational interactions with various P2 residues, particularly at positions 36–38 (Fig. 5a). We also observed rescue of mutations at positions 36 and 37 by mutations in C61, which normally basepairs with G38 (Fig. 5a). We suspect these rescues are all associated with various structural rearrangements of P2, potentially including more substantial changes involving residues in its adjacent 39–42 and 57–60 linker elements. The *glmS* ribozyme would appear to be particularly tolerant of mutations in P2, due to this element’s propensity for structural rearrangement. As a corollary, we anticipate that the P2 sequence would show significant drift over evolutionary time. Given that P2 appears to be a structurally important element with no direct role in catalysis, flexibility in its rearrangements might confer a selective advantage in the face of mutational load.

### Certain residues exhibit widespread mutational interactions

Mutation A8G in the P1 hairpin base was able to rescue second-site mutations throughout the ribozyme (Fig. 5a). While A8G alone is ~1.4-fold more active than the consensus sequence, the minor increase in baseline activity does not explain the observed rescues, many of which are more substantial (e.g., an eightfold rescue of the highly detrimental C4G mutation). A8G presumably still forms a basepair with its consensus partner, U25. We speculate that the resulting G•U wobble pair generated at the base of P1 may lead to a more stable fold that is more resistant to active-site destabilization by other mutations, compared to the consensus sequence.

Mutations in the bulged P2.2 nucleotide, A6, were also found to rescue mutations at various positions throughout the ribozyme (Fig. 5a), and A6U, in particular, rescued multiple second-site mutations. Similar to A8G, A6U exhibited ~1.5-fold higher catalytic activity than the consensus sequence, but this modest improvement in baseline activity does not fully account for the rescued activity achieved. As noted previously, A6U is conjectured to rescue U24C (in the P1 base) by forming a tertiary contact with the 9•24 basepair. However, A6U is also able to rescue mutations at more distal locations, such as C50A (by approximately fourfold). We speculate that A6U, like A8G, may stabilize the ribozyme fold in some manner that reduces the severity of many other mutations. The predicted interaction between the 9•24 basepair and a flipped-in base at A6U (Fig. 5c) might be the mechanism for such stabilization. For both A8G and A6U, such potentially stabilizing interactions involve the base of the P1 hairpin. We observed two further classes of mutants (C44G in P2.1, and purine substitutions of C62, U63, or C64 in P2) that are also able to rescue mutations situated throughout the ribozyme (Fig. 5a). Unlike A8G and A6U, these two classes seem to generate rescues with generally similar cleavage rates, regardless of the identity of the second mutation (10^−5^ – 10^−4^ s^−1^; Fig. 4a). We investigated these unusual mutants further (Supplementary Discussion, Supplementary Fig. 5).

## Discussion

The measurement of reaction kinetics supplies a powerful tool for characterizing enzymes and ribozymes. When carried out with the wild-type molecule and sufficiently large numbers of mutated variants, such measurements can shed light on the roles played by individual residues, as well as by interactions among them. Comprehensive functional maps that assess the impact of all possible point and pairwise mutations, for example, can improve our understanding of sequence–structure–function relationships and guide efforts to engineer novel biocatalysts. In addition, comparisons of such maps with other systematic datasets, such those reporting natural sequence conservation^9^, or selection fitness in vitro^32–35^ or in vivo^18,19^, can suggest mechanisms that underlie the higher-order effects of sequence variations. In practice, the sheer number of measurements required to score the kinetics of all single and double mutants surpasses the scale accessible to traditional biochemistry. The approach described here solves this problem for a self-cleaving ribozyme by recording reaction kinetics in a massively parallel manner, using a fluorescence signal derived from synthetic RNA variants bound in clusters to a sequencing chip.

Quantities such as the binding affinity, dissociation constant, and reaction rate serve to characterize biochemical function. In the case of riboswitches, the important control parameters are (1) the maximum switching activity and (2) the cognate ligand sensitivity. For the *glmS* riboswitch, these parameters are determined by the values of *k*_cat_ and *K*_M_, respectively. Ribozyme *k*_cat_ and *K*_M_ values were derived from real-time records of self-cleavage kinetics, scored for each sequence variant at multiple ligand concentrations (apparent *K*_M_ values were determined at physiological magnesium concentrations; see Methods). Comparisons of the functional effects of mutations with natural sequence variation among the bacterial ribozymes^9^ shed light on how evolutionary selection may have acted on these parameters. The array data revealed a striking disparity in the variability of kinetic parameters among functional variants. Most point mutations caused only a small (less than two-fold) or negligible change in the apparent *K*_M_, whereas many of these same mutations produced hundred-fold or even larger effects on *k*_cat_. The frequency of natural point mutations was found to be strongly correlated with the widely dispersed *k*_cat_ values of the corresponding variants, but not with their apparent *K*_M_ values (Fig. 2). These findings suggest that sequence conservation is driven primarily by the catalytic rate achieved, and not by the ligand-sensitivity.

Double mutations can serve to map interactions between residues, for instance, via double-mutant rescue or epistasis. Deep mutational scanning studies have revealed pairwise interactions in a number of different systems, including various proteins^36–38^ and a snoRNA^18^. Pairwise mutational interactions were likewise observed in RNA array experiments on protein binding to nucleic acid variants^25^. The rescue heatmap of *glmS* double mutants revealed numerous such pairwise interactions (Fig. 5). Strikingly, this heatmap successfully identified, at nucleotide resolution, all interactions associated with secondary structural elements in the ribozyme. Comparisons between the pairwise rescue heatmap and the corresponding map of cleavage rates (Fig. 4) reveal the relative structural and catalytic contributions of base-paired residues, that is, those that serve mainly a structural role (where any alternative basepairs would maintain activity) and those that play more direct roles in catalysis (where non-consensus basepairs were functionally deficient). The method reported here offers a general approach to combined structure-function mapping of ribozymes, even those presently unknown at atomic resolution, complementing existing mapping approaches based on accessibility to chemical modification^39–42^.

Double-mutant interactions supply insights not only into structural features of the consensus ribozyme, but also into structural rearrangements that occur in certain mutant contexts. For example, the rescue heatmap supplies numerous examples of presumptive realignments in duplex elements that minimize basepair mismatches, including bulge-shifting. In other cases, the data suggest the presence of novel tertiary contacts that may form to rescue activity. The existence of such postulated structures remains to be verified, and offers opportunities for further exploration. The rescue data also revealed that certain mutations (e.g., A6U, A8G, and (62–64)R) engage in multiple interactions with residues situated throughout the structure. The mechanisms responsible for such promiscuous interactions are presently unknown, and suggest new lines of inquiry.

No variant in the library was found to cleave substantially faster than the consensus rate. This finding implies that the core ribozyme is located near a local optimum in the space of sequences, raising two possibilities. The first is that the core ribozyme architecture cannot support substantially higher cleavage rates when constrained by the twin evolutionary requirements of ligand dependence and ligand specificity. The second possibility is that higher cleavage activity is, in fact, accessible by mutation, but that evolution has selected a lower-activity solution: one located at least three mutations away from substantially more active variants. The latter possibility might arise, for example, from constraints on the feedback loop mediated by the riboswitch. Excessively high cleavage rates may be detrimental to fitness^43,44^, owing to insufficient levels of the GlcN6P cell wall precursor, and experiments with artificial *glmS* ribozyme activators have shown that inappropriately high cleavage activity can be lethal for bacteria^45^.

In summary, by assaying a full set of single and double mutants of the *glmS* ribozyme on an RNA array, we successfully mapped the catalytic consequences of sequence variation in detail. This approach enables a systematic investigation of how specific biochemical parameters, such as the catalytic rate and the Michaelis constant, can drive sequence conservation in functional nucleic acids of roughly 150 or fewer residues, where an exhaustive search of local mutational space is now feasible. Double-mutant rescue heatmaps facilitate a new approach to structural (and functional) mapping with nucleotide-level resolution, and can point to the presence of novel structural rearrangements. We believe that this approach and similar high-throughput methods will have broad utility in characterizing a variety of other functional nucleic acids.

## Methods

### Sequencing library

The DNA sequences used for constructing the sequencing library are listed in Supplementary Table 2. The library layout is shown in Fig. 1b, c. Library construction began by combining a core *glmS* ribozyme-encoding sequence (consisting of the “construct 1” sequence previously characterized by Soukup^24^ plus additional flexible linkers, reaching 5 nt upstream and 3 nt downstream) with an upstream partial *E. coli* RNA polymerase (RNAP) stall sequence (controlled by nucleotide deprivation), and a downstream Illumina primer (Read2) sequence. The resulting glmS_core oligomer was synthesized with a 1.3% mole fraction of each non-consensus nucleotide at the underlined positions (Supplementary Table 2): this doping rate was selected to maximize the numbers of sequences in the final pool carrying two mismatches, assuming Poisson statistics for synthesis. The glmS_core oligomer library was then extended by PCR to add Illumina sequencing adapters, a 16-nt random barcode, and an RNAP promoter plus a complete RNAP stall sequence (Fig. 1c). This extension was performed with the oligomer pool (glmS_core; 1.5 nM), long flanking DNAs (C_R1_BC_RNAPall and D_read2; 3.8 nM), and outer primers (C_adapter and D_adapter; 137 nM). The sequencing library was bottlenecked by dilution to ~700,000 molecules. Bottlenecking allows a smaller population of unique molecules to be amplified, and thus limits the total number of distinct molecular variants that are assayed on the RNA array system in order to generate multiple measurements (across multiple clusters) for each variant^25^. After bottlenecking, we carried out PCR amplification with both C_adapter and D_adapter. The library was then quantified for sequencing by qPCR and sequenced on several different chips on an Illumina MiSeq device.

### Data collection

Following sequencing on the MiSeq device, chips were mounted on custom fluorescence microscopes^25^ for imaging. dsDNA templates were generated by removing residual complementary DNA strands and fluorophores via denaturation with heat and formamide, hybridizing a biotinylated oligomer (D_read2_biotin) to the end of the ssDNA sequences, and extending the template using Klenow Fragment (3′→5′ exo–) (NEB). A biotin-streptavidin RNAP roadblock was created by incubating with 1 μM streptavidin followed by 5 μM biotin (7 min each) (Fig. 1c).

RNA was generated in situ from the DNA clusters on-chip. *E. coli* RNAP (NEB) was introduced in RNAP buffer (20 mM Tris-HCl pH 7.5, 7 mM MgCl_2_, 20 mM NaCl, 0.0973% BME, 0.1 mM EDTA, 1.5% glycerol, 20 μg/ml BSA, 0.01% Tween-20), with 2.5 μM ATP, GTP, and UTP, but no CTP, allowing it to initiate at the RNAP promoter sequence but stall at the first C residue of the stall sequence. Excess RNAP was then removed, followed by extension with 1 mM each of all four NTPs, in either RNAP buffer or HEPES-RNAP buffer (50 mM HEPES pH 7.5, 20 mM KCl, 7 mM MgCl_2_, 0.0973% BME, 0.1 mM EDTA, 1.5% glycerol, 20 μg/ml BSA, 0.01% Tween-20) with 500 nM of each fluorescent probe oligomer (Cy5_Read2 and RNAPstall_prime_Cy3). Transcription was performed at 37 °C.

Following 5 min transcription with all four NTPs and both fluorescent probe oligomers present, the chip was equilibrated for 10 min in cleavage buffer (50 mM HEPES pH 7.8, 150 mM KCl, 4 mM Mg(OAc)_2_, 100 μM Na_2_EDTA, 1 mM DTT) containing 500 nM of each fluorescent probe oligomer, and then ~5 min in cleavage buffer without fluorescent oligomers. Next, the chip was imaged twice to establish a baseline at *t* = 0, following which cleavage buffer with glucosamine-6-phosphate (GlcN6P) (10 mM, 2.5 mM, 640 μM, 160 μM, or 40 μM) was introduced while the chip was imaged. The flow was initially fast (150 μL/min for ~5 min), then maintained at a slower rate (15 μL/min) to remove cleaved RNA hybridized to fluorescent oligomers. Imaging was initially done continuously on three out of nineteen tiles on the sequencing chip, to capture faster kinetics, followed by more tiles and longer times between imaging, for a total experimental run-time of ~14 h. All experiments were performed at 37 °C.

### Data analysis

The raw fluorescence images were aligned to the sequencing data and quantified using the green channel signal from the Cy3-labeled RNAPstall_prime_Cy3 oligomer^25^. Sequences from seven separate chips were pooled to determine and extract unique molecule identifiers (barcodes) for the sequences. For each barcode, a consensus sequence was determined, based on the most common base at each position. Barcodes were kept when at least 66% of sequences matched this consensus at each position, and the *p* value for each position was below 0.05, based on a binomial test. For the analysis of fluorescence experiments, sequences were used if the Hamming distance between the sequence and its corresponding barcode consensus sequence was <3.

Fluorescence intensities for each cluster at all time points were first normalized to the average intensity obtained from two images taken prior to the introduction of GlcN6P. To account for the combined effects of photobleaching, RNA degradation, possible drift in focus, and other systematic variations, cluster intensities were further normalized to the numerical average of the median intensities recorded for each of ten non-cleaving single mutants (G1C, G1U, C2G, C2A, G3C, A27G, C28G, G56C, G56U, G57C), for each tile and time point. The time series of normalized per-tile median values for each variant was then fit to one- and two-rate models for cleavage using the Levenberg–Marquardt algorithm implemented in Python^46^. Constraints were imposed for minimum and maximum values, with constraint values appropriate for the individual experiment. Single- and double-exponential fits were considered for each variant dataset, and the better fit was determined using the Bayesian Information Criterion (BIC), calculated in Python^46^. A single exponential was adopted unless the double-exponential fit improved the BIC by at least 10 units, corresponding to “very strong” evidence against the single exponential model^47^. When the better fit was a double exponential, the faster rate was taken to represent characteristic cleavage rate, so long as it represented at least 10% of the total decay amplitude; otherwise, the slower rate was adopted. All fits were quality-filtered using a maximum BIC threshold. Weighted fits to the Michaelis–Menten model, $k_{{\mathrm{obs}}}\left( {\mathrm{C}} \right) = k_{{\mathrm{cat}}}{\mathrm{C}}/\left( {K_{\mathrm{M}} + {\mathrm{C}}} \right)$, where C = [GlcN6P]), were then performed for each variant, using the best-fit rates (*k*_obs_) and associated parameter errors [σ(*k*_obs_)] for the five measured GlcN6P concentrations (0.04, 0.16, 0.64, 2.5, and 10 mM). Only *k*_cat_ and *K*_M_ values wherein σ(*k*_cat_) < *k*_cat_ and *σ*(*K*_M_) < *K*_M_, respectively, were accepted. The double-mutant rescue plot was generated as follows. For each double-mismatch mutant (*i*, *j)*, and reference single mutant *j*, the mutational rescue was calculated as *k*_obs_(*i, j*)/*k*_obs_(*j*), where *j* is the single mutant along the abscissa of the rescue plot. Only values > 1, indicating rescue, were considered: other values were set to a background of 1 for the heatmap. Rescue values were only accepted as significant when *k*_obs_(*i, j*) was separated from *k*_obs_(*j*) by ≥1σ; in isolated cases where the fit parameter error could not be determined for the single mutant, or where *k*_obs_(*j*) ≤ 1 × 10^−6^ s^−1^ (below the minimum detectable rate), $\sigma \left( {k_{{\mathrm{obs}}}(i,j)} \right)$ was used to assess the difference.

### Analysis of biological sequences

Sequences were downloaded from NCBI using the Efetch utility, based on the accession numbers and nucleotide positions supplied in the Supplemental section of ref. ^9^. In all, 385 unique sequences were successfully compiled for analysis. These sequences were aligned to the *glmS* ribozyme consensus using the Needleman–Wunsch algorithm (EMBOSS Needle), and each stem or loop segment of the ribozyme was assigned after further alignment. A short list of 38 sequences was excluded from our analysis due to updated reference genomes, partial sequence data, absence of expected stems, or no obvious match with the consensus sequence. The per-base conservation frequencies were calculated from the ratio of the numbers of each mutation scored to the total number of sequences with available data at each base position.

### Gel-based measurements of self-cleavage activity

Ribozyme activity in bulk solution was assayed using an RNA sequence coding for the core ribozyme with short 5′ and 3′ linker sequences attached (AUAAA and AAA, respectively) matching those found in the on-chip construct (oligomer glmS_core_gel in Supplementary Table 2). RNA oligomers corresponding to each sequence variant assayed were generated by solid-phase synthesis (Integrated DNA Technologies). Reactions were performed as follows. The RNA was prepared in a solution of cleavage buffer, denatured at 80 °C for 2 min, and then incubated for 30 min at 37 °C to allow time for the slow refolding step to go to completion^28^. GlcN6P, dissolved in cleavage buffer, was then added to a final concentration of 10 mM. For reactions without GlcN6P, an appropriate volume of cleavage buffer was added instead. Reactions were performed at 37 °C unless noted otherwise. Individual reactions were stopped at the desired time by adding an equal volume of stop buffer containing 95% formamide and 18 mM EDTA (Gel Loading Buffer II, Thermo Fisher), mixing, and immediately placing the reaction on ice or at −20 °C. Reaction products were subsequently incubated at 95 °C for 5 min and run on 15% denaturing PAGE gels (Mini-Protean TBE-Urea, Bio-Rad). Gels were stained with SYBR Gold (Thermo Fisher) and imaged with a UV transilluminator. For kinetics, the fraction cleaved was determined from the ratio of intensity of the band for the 3′ cleavage product to that of the uncleaved band, measured using ImageJ. This fraction cleaved, *F*(*t*), was plotted and fit to the double exponential expression

1 $$F\left( t \right) = F\left( 0 \right) + \left[ {F_{{\mathrm{max}}} - F(0)} \right]\left\{ {1 - \left[ {f_{{\mathrm{fast}}}\exp \left( { - k_{{\mathrm{fast}}}t} \right) + \left( {1 - f_{{\mathrm{fast}}}} \right){\mathrm{exp}}( - k_{{\mathrm{slow}}}t)} \right]} \right\}$$

using Igor Pro (Wavemetrics).

### Reporting summary

Further information on research design is available in the Nature Research Reporting Summary linked to this article.

## Supplementary information

Supplementary Information

Reporting Summary

## Data Availability

Source data for the figures and supplementary figures are provided as a Source Data file. The Source Data file includes a document which functions as a guide for connecting various source data to their corresponding figures. Other data supporting the findings of this study are available from the corresponding authors upon reasonable request.

## Source Data

Source Data file
