# Supplementary material for: Comprehensive sequence-to-function mapping of cofactor-dependent RNA catalysis in the glmS ribozyme
Source: Nat Commun. 2020 Apr 3;11:1663. doi: 10.1038/s41467-020-15540-1 (PMC7125110; doi:10.1038/s41467-020-15540-1)
Supplement: Supplementary file 1 — Supplementary Information [file 41467_2020_15540_MOESM1_ESM.pdf]

Supplementary Information for:

Comprehensive sequence-to-function mapping of cofactor-dependent RNA catalysis in the *glmS* ribozyme

J.O.L. Andreasson and A. Savinov *et al.*

Supplementary Figures:

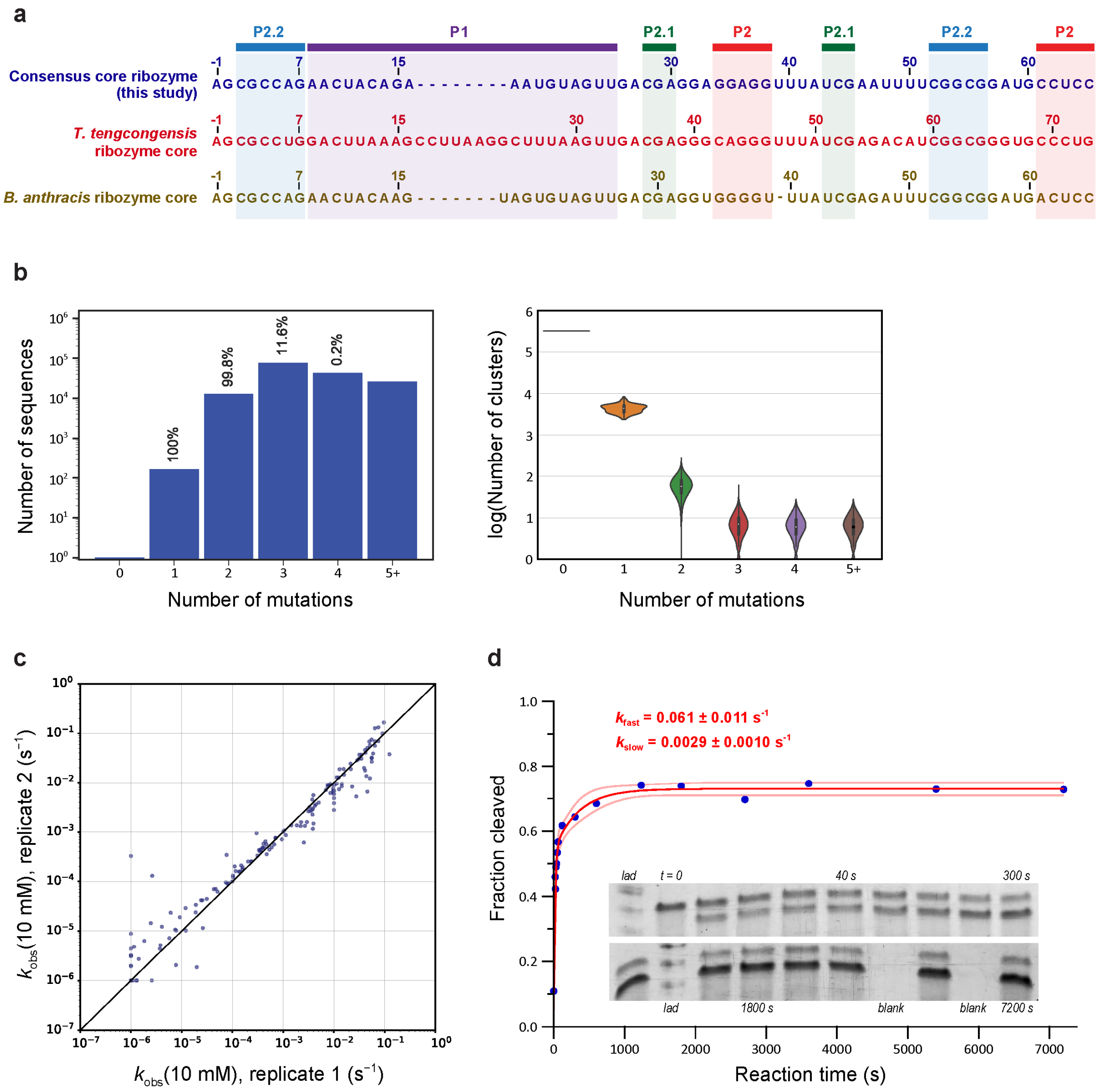

Supplementary Figure 1. Library properties, assay reproducibility, and comparison to gel-based measurements. **a**, Sequence alignment of the core ribozyme construct investigated here (ref. 1) with the core domains of the *T. tengcongensis* (crystal structure shown in Fig. 1a, refs. 2,3) and *B. anthracis* (ref. 4) *glmS* ribozymes. Secondary structure elements are indicated as in Fig. 1b. **b**, Distribution of mutation numbers for variants. Left: numbers of sequence variants present in the library carrying each possible number of mutations. Right: Violin plot of the number of clusters for each type of mutation on a typical sequencing chip. **c**, Reproducibility: comparison of two measurements of single mismatch mutant cleavage rates at 10 mM GlcN6P on two different sequencing chips. The line shows $x = y$. The geometric mean difference in rate between any two replicates is 1.6-fold. Source data are available in the Source Data file. **d**, Self-cleavage kinetics of the core ribozyme, derived from gel measurements [Methods]. Cleavage reactions were performed for the indicated time with 10 mM GlcN6P at 25 °C. The fraction cleaved was fit to an expression for double exponential decay, $F(t) = F(0) + [F_{max} - F(0)]\{1 - [f_{fast}\exp(-k_{fast}t) + (1 - f_{fast})\exp(-k_{slow}t)]\}$. The fit curve (red line) is plotted with 95% confidence intervals (tan lines). Fit parameters: $k_{fast} = 0.061 \pm 0.011$ s^{-1}, $k_{slow} = 0.0029 \pm 0.0010$ s^{-1}, $f_{fast} = 0.69 \pm 0.05$, $F_{max} = 0.73 \pm 0.01$, and $F(0) = 0.11 \pm 0.02$ (values ± σ). Inset: gel image from which these values were derived.

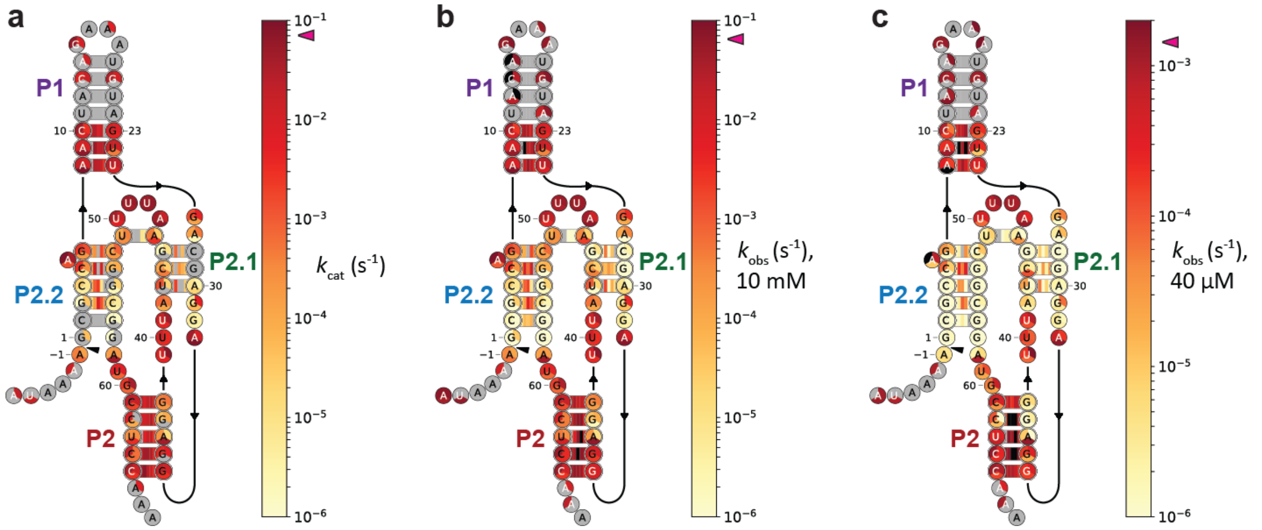

Supplementary Figure 2. Saturating- and subsaturating-condition cleavage rates compared to k_{cat}. **a**, Structural heatmap of k_{cat}, compared with similar heatmaps for **b**, k_{obs}(10 mM GlcN6P) and **c**, k_{obs}(40 µM GlcN6P), for all single mutants and basepaired-partner double mutants. The color map for **c** has been rescaled to emphasize the similar trend in cleavage rate reduction. Source data are available in the Source Data file.

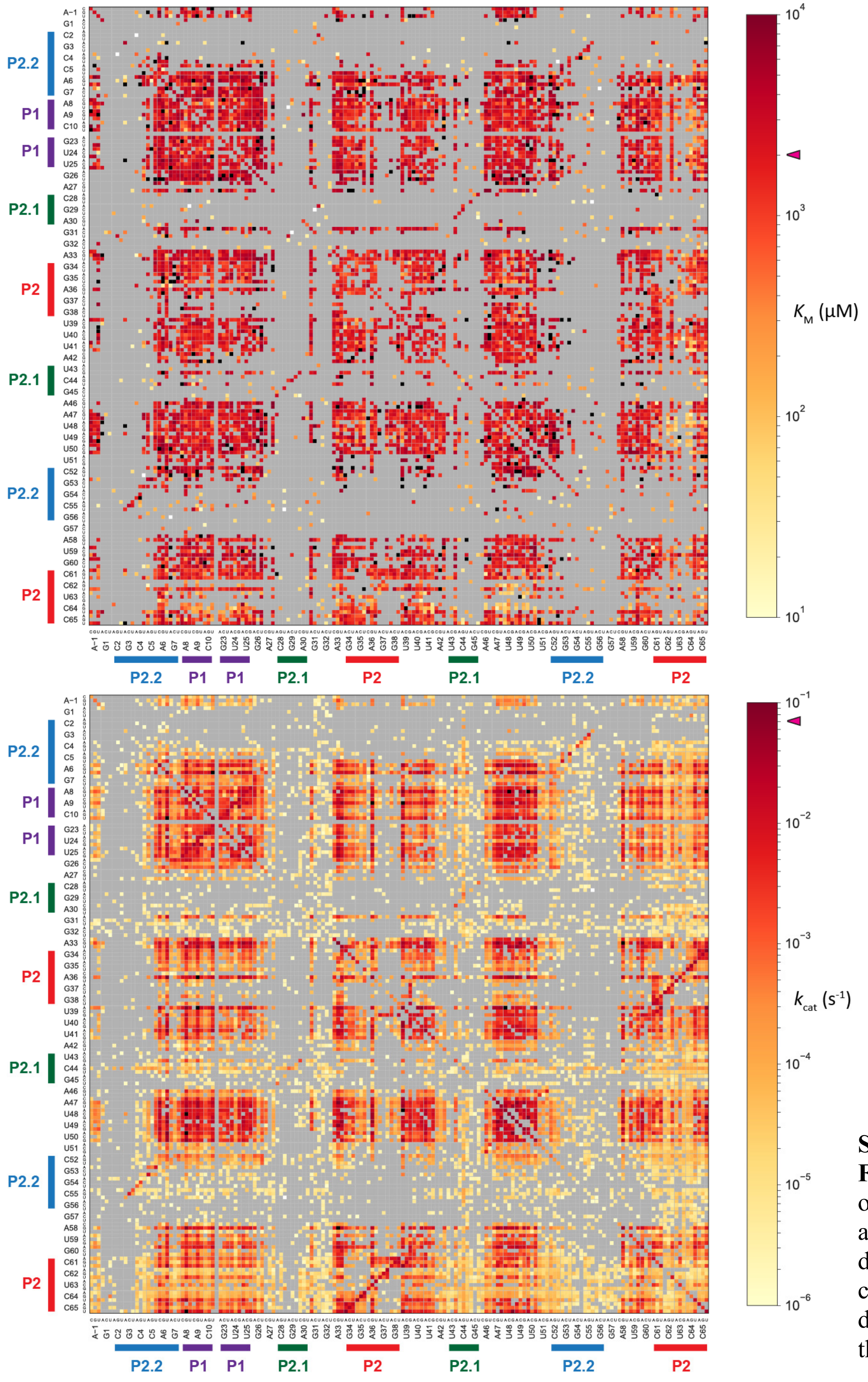

Supplementary Figure 3. Heatmaps of k_{cat} and apparent K_M across all single and double mutants of the core ribozyme. Source data are available in the Source Data file.

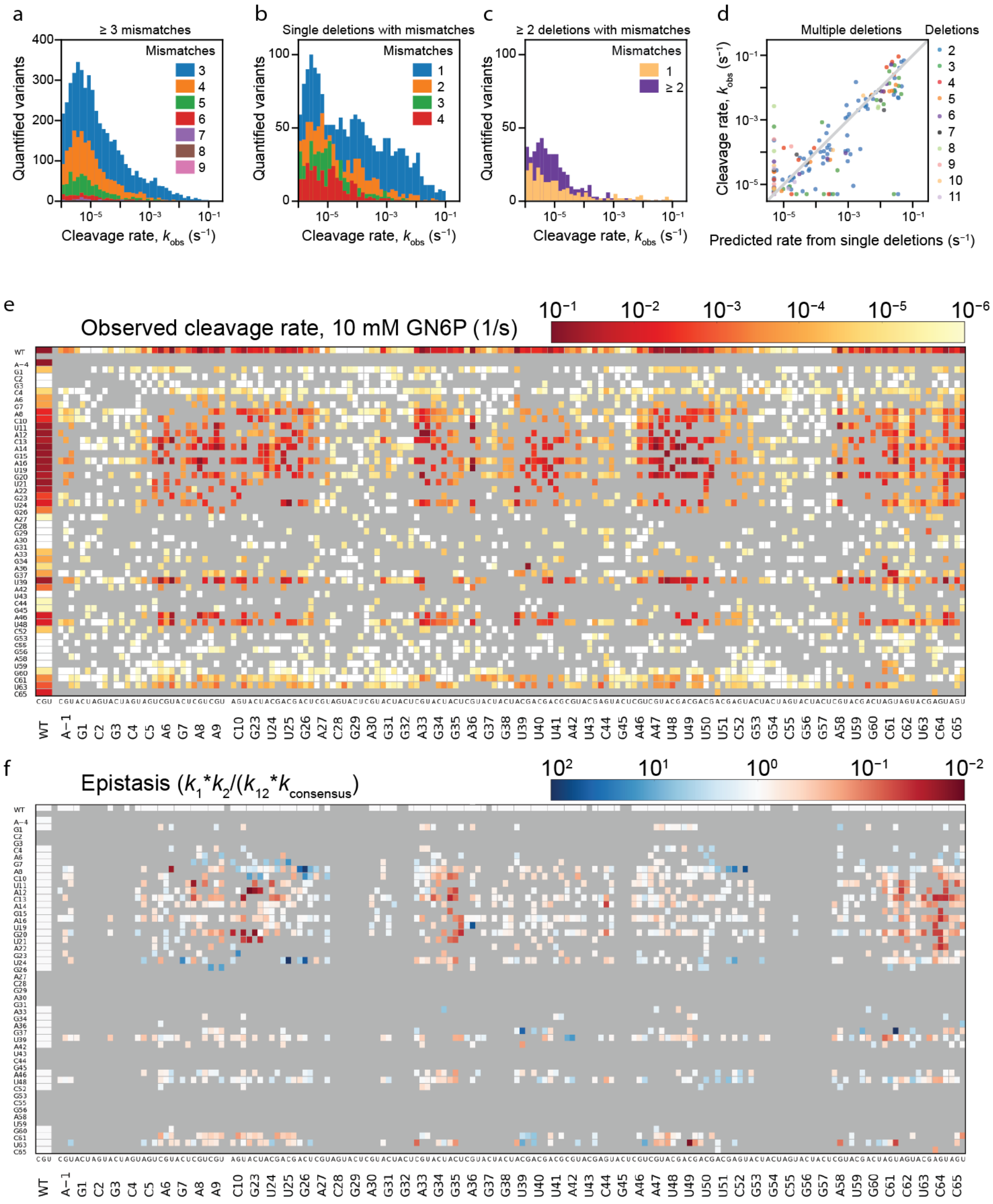

Supplementary Figure 4. Quantification of higher-order mutations. **a**, Histogram of k_{obs}(10 mM) for variants with three or more mismatches. **b**, Histogram of k_{obs}(10 mM) for variants with a single deletion and one or more mismatches. **c**, Histogram of k_{obs}(10 mM) for variants with two or more deletions in combination with one or more mismatches. **d**, k_{obs}(10 mM) for variants with two or more deletions (no mismatches) plotted vs. rates predicted from the corresponding single deletions. The predicted rate for was calculated as $k_{mutant} \times \sum k_i / k_{consensus}$ and the minimum measured or predicted rate was set to 5×10^{-6} s^{-1}. **e**, Heatmap of k_{obs}(10 mM) for variants with one mismatch (*x*-axis) and one deletion (*y*-axis). **f**, Heatmap of k_{obs}(10 mM) epistasis for variants with one mismatch (*x*-axis) and one deletion (*y*-axis). Epistasis was defined the ratio of the combined impact of the single mutations, relative to the consensus sequence, over the impact of the double mutation, also relative to the consensus sequence, i.e.,

$$Epistasis = (k_2/k_{consensus})(k_2/k_{consensus})/(k_{12}/k_{consensus}) = k_1 k_2/(k_{12} k_{consensus}).$$

Positive epistasis (blue squares) indicates sequences with higher activity than predicted from the single mutations. Source data are available in the Source Data file.

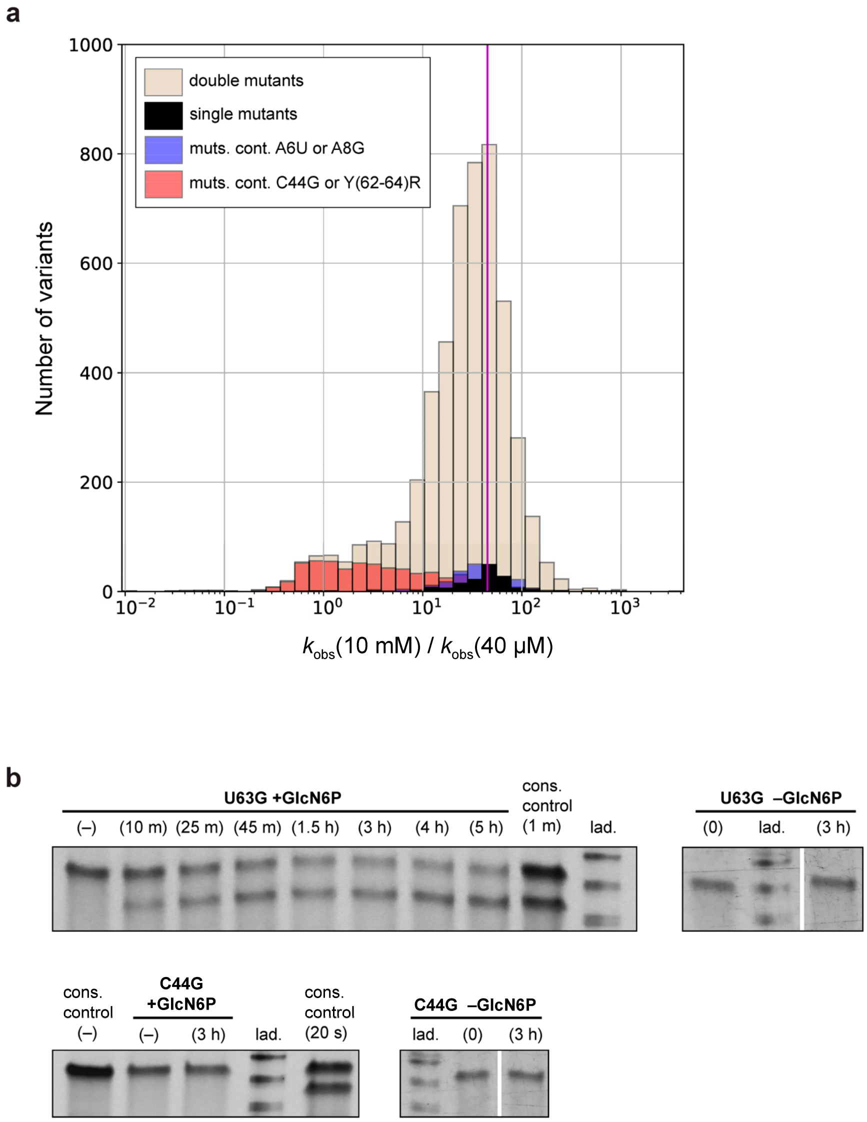

Supplementary Figure 5. Further investigation of mutations that display ribozyme-wide rescue interactions. **a**, Histogram of k_{obs}(10 mM) / k_{obs}(40 μM) for k_{obs}(10 mM) > 1×10^{-5} s^{-1}. The magenta line indicates the consensus sequence value of the cleavage rate ratio. Source data are available in the Source Data file. **b**, Bulk self-cleavage measurements performed on U63G and C44G ribozymes. All +GlcN6P measurements were performed with 10 mM of the cofactor; "–" indicates the initial t = 0 time point, immediately prior to GlcN6P addition. Outlines delineate bands from same gel. A single-exponential fit to the fraction cleaved as a function of time from the U63G +GlcN6P gel experiments yielded a cleavage rate of $(1.0 \pm 0.3)\times10^{-3}$ s^{-1}, within ~2-fold of the on-chip measured rate $(4.7 \pm 0.8)\times10^{-4}$ s^{-1}.

Supplementary Discussion:

The P1 hairpin base has little effect on catalysis when mutated. The covariation of nucleotides at the base of P1[5] (Fig. 2c) suggests that the stability of this hairpin base may be of functional importance. One possibility is that a stably folded P1 base is important for forming a tight junction between P1 and P2.2/P2.1, which could be an important geometric constraint for active site formation. However, we find that single point mutations causing mispairing in the bottom three basepairs of P1 do not strongly affect catalytic activity (Fig. 2a, Supplementary Figure 2b), with a log-average rate reduction of 8-fold. (This is in spite of the notable exception of U24C, which causes a ~160-fold rate reduction.) Even double mutants which unpair both bottom basepairs of P1 can have consensus-like rates, e.g., k_{obs}(10 mM) = 2.6×10^{-2} s^{-1} for U24A,U25A, and the log-average rate reduction for doubly-mispaired P1 base mutants is 37-fold. Interestingly, P1 base-unfolding double mutants in the 3′ strand of the hairpin are particularly well-tolerated: doubly-unpaired U24X,U25X mutants cause a log-average rate reduction of only 7-fold. Overall, then, mutations that destabilize the base of P1 tend to be well-tolerated when it comes to catalysis, suggesting that the tightness of the junction between P1 and P2.2/P2.1 is not catalytically critical. We hypothesize that the covariation of these basepairs in bacterial sequences is instead due to effects of mutations in this region on the ribozyme folding rate. This is consistent with P1 nucleating folding of the P2.2-P2-P2.1 double pseudoknot, helping bring together the distal sequence elements involved in the fully folded structure[6].

Therefore, our RNA array measurements allow us to not only determine major mechanisms underlying sequence conservation, but also to rule out particular mechanisms for conservation of specific sequence features. In the case of G23 and P1 hairpin base mutations of the *glmS* ribozyme, it is clear that sequence conservation is driven by factors other than cleavage rate.

Discussion of double mutants containing C44G and (62–64)R. The relative insensitivity of C44G- and (62–64)R-containing double mutants to the identity of the second-site mutation suggests that these particular mismatches may produce an altered ribozyme structure. Such mutants might represent ligand-independent self-cleaving ribozymes, or ribozymes with substantially reduced K_M. Indeed, double-mutant variants containing (62–64)R and C44G (but notably, not A8G or A6U) tend to exhibit similar cleavage rates in 40 μM and 10 mM GlcN6P (Supplementary Figure 5a). However, these mutants' insensitivity to second-site mutations and cofactor concentration is also consistent with an alternative hypothesis: namely, that for such variants, observed slow fluorescence decay is due to a misfolding process rather than self-cleavage (e.g., formation of an alternative RNA structure that quenches Cy3 or outcompetes binding of the Cy3-labeled oligomer). We thus investigated self-cleavage activity of representative mutants U63G and C44G in gel experiments. These experiments revealed that C44G exhibits no cleavage even after 3 hrs in saturating (10 mM) GlcN6P (Supplementary Figure 5b), suggesting that mutants of the C44G class undergo a misfolding process as outlined above. On the other hand, our bulk experiments with U63G reproduce ligand-stimulated cleavage, and show that U63G lacks self-cleavage activity in the complete absence of cofactor ligand (Supplementary Figure 5b). Thus mutants of the (62–64)R class have markedly reduced cofactor-dependence compared to the consensus ribozyme, at the price of substantially lower saturating activity.

Higher-order mutations. Our sequence dataset sparsely sampled higher-order mutations (≥3 mismatches, ≥2 deletions, as well as combinations of mismatches and deletions). Kinetics were quantified for 20983 of these sequences, and 58% (12136 variants) showed self-cleavage rates $>10^{-6}$ s^{-1} (Figs S5a-S5f). A large number (927) cleaved relatively fast ($>10^{-3}$ s^{-1}), including variants with up to eight mismatches throughout their sequence (Fig S5a). Up to eight deletions

could also be tolerated, mainly in the P1 stem, indicating that a truly minimal *glmS* ribozyme is made up of fewer nucleotides than the consensus core construct (Fig S5d).

Combinations of deletions and mismatches with self-cleavage activity were common (>4000 variants, Fig S5b). Deletion-mismatch double mutants were covered fairly well; a heatmap of the rates of these mutants is shown in Fig S5e. By comparing observed rates with those predicted by multiplicatively combining single deletion and single mismatch rates (epistasis heatmap, Supplementary Figure 4f), many instances can be identified where deletions rescue otherwise detrimental mutations. For example, mutations in A42 (A42C or A42G) are rescued by deleting the preceding base. The converse is also observed, where rather harmless deletions and mismatches together render the ribozyme nearly inactive, even when the mutated residues are far apart.

Supplementary Tables:

Supplementary Table 1. Correlations of mutation frequency (f_{mut}) with k_{obs} at various GlcN6P concentrations.

**[GlcN6P], uM**	**Spearman's *ρ***	***P* (Spearman's, two-tailed t-test)**
10,000	0.796	9.7×10^{-37}
2,500	0.779	3.3×10^{-34}
640	0.795	1.3×10^{-36}
160	0.776	8.6×10^{-34}
40	0.767	1.3×10^{-32}

Supplementary Table 2. Oligonucleotide sequences used in this study. Underlined bases indicate positions that were mutagenized by doped synthesis.

**glmS_core**	tgtatggaagacgttcctggatccATAAAAGCGCCAGAACTACAGAAATGTAG<br>TTGACGAGGAGGAGGTTTATCGAATTTTCGGCGGATGCCTCCAAA<br>agatcggaagagcggttcag
**glmS_core_gel**	AUAAAAGCGCCAGAACUACAGAAAUGUAGUUGACGAGGAGGAGG<br>UUUAUCGAAUUUUCGGCGGAUGCCUCCAAA
**C_R1_BC_RNAPall**	AATGATACGGCGACCACCGAGATCTACACTCTTTCCCTACACGAC<br>GCTCTTCCGATCTNNNNNNNNNNNNNNNNNNTTTATGCTATAATTATT<br>TC ATGTAGTAAGGAGGTTGTATGGAAGACGTTCCTGGAT
**D_read2**	CAAGCAGAAGACGGCATACGAGATCGGTCTCGGCATTCCTGCTGA<br>ACCGCTCTTCCGATCT
**D_read2_biotin**	/5bio/CAAGCAGAAGACGGCATACGAGATCGGTCTCGGCATTCCTG<br>CTGAACCGCTCTTCCGATCT
**C_adapter**	AATGATACGGCGACCACCGAGATCTACAC
**D_adapter**	ATCTCGTATGCCGTCTTCTGCTTG
**RNAPstall_prime**	GGATCCAGGAACGTCTTCCATACAACCTCCTTACTACAT
**RNAPstall_prime_Cy3**	GGATCCAGGAACGTCTTCCATACAACCTCCTTACTACAT/3Cy3/
**Cy5_Read2**	/5Cy5/CAAGCAGAAGACGGCATACGAGATCGGTCTCGGCATTCCT<br>GCTGAACCGCTCTTCCGATCT

Supplementary References:

1. Soukup, G.A. Core requirements for glmS ribozyme self-cleavage reveal a putative pseudoknot structure. *Nucleic Acids Res.* **34**, 968-975 (2006).
2. Klein, D.J. & Ferre-D'Amare, A.R. Structural basis of glmS ribozyme activation by glucosamine-6-phosphate. *Science* **313**, 1752-1756 (2006).
3. Klein, D.J., Wilkinson, S.R., Been, M.D. & Ferre-D'Amare, A.R. Requirement of helix p2.2 and nucleotide g1 for positioning the cleavage site and cofactor of the glmS ribozyme. *J. Mol. Biol.* **373**, 178-189 (2007).
4. Cochrane, J.C., Lipchock, S.V. & Strobel, S.A. Structural investigation of the GlmS ribozyme bound to its catalytic cofactor. *Chemistry & Biology* **14**, 97-105 (2007).
5. McCown, P.J., Roth, A. & Breaker, R.R. An expanded collection and refined consensus model of glmS ribozymes. *RNA* **17**, 728-736 (2011).
6. Savinov, A. & Block, S.M. Self-cleavage of the glmS ribozyme core is controlled by a fragile folding element. *Proc. Natl Acad. Sci. USA* **115**, 11976-11981 (2018).
