# Supplementary material for: Comprehensive sequence-to-function mapping of cofactor-dependent RNA catalysis in the glmS ribozyme
Source: Nat Commun. 2020 Apr 3;11:1663. doi: 10.1038/s41467-020-15540-1 (PMC7125110; doi:10.1038/s41467-020-15540-1)
Supplement: Supplementary file 2 — Reporting Summary [file 41467_2020_15540_MOESM2_ESM.pdf]

nature research

Corresponding author(s): William Greenleaf and Steven Block

Last updated by author(s): 02/05/2020

Reporting Summary

Nature Research wishes to improve the reproducibility of the work that we publish. This form provides structure for consistency and transparency in reporting. For further information on Nature Research policies, see Authors & Referees and the Editorial Policy Checklist.

Statistics

For all statistical analyses, confirm that the following items are present in the figure legend, table legend, main text, or Methods section.

n/a	Confirmed	
☐	☒	The exact sample size (*n*) for each experimental group/condition, given as a discrete number and unit of measurement
☐	☒	A statement on whether measurements were taken from distinct samples or whether the same sample was measured repeatedly
☐	☒	The statistical test(s) used AND whether they are one- or two-sided<br>*Only common tests should be described solely by name; describe more complex techniques in the Methods section.*
☒	☐	A description of all covariates tested
☒	☐	A description of any assumptions or corrections, such as tests of normality and adjustment for multiple comparisons
☐	☒	A full description of the statistical parameters including central tendency (e.g. means) or other basic estimates (e.g. regression coefficient) AND variation (e.g. standard deviation) or associated estimates of uncertainty (e.g. confidence intervals)
☐	☒	For null hypothesis testing, the test statistic (e.g. F, t, r) with confidence intervals, effect sizes, degrees of freedom and P value noted<br>*Give P values as exact values whenever suitable.*
☒	☐	For Bayesian analysis, information on the choice of priors and Markov chain Monte Carlo settings
☒	☐	For hierarchical and complex designs, identification of the appropriate level for tests and full reporting of outcomes
☒	☐	Estimates of effect sizes (e.g. Cohen's *d*, Pearson's *r*), indicating how they were calculated

Our web collection on statistics for biologists contains articles on many of the points above.

Software and code

Policy information about availability of computer code

Data collection	Data was collected using a repurposed Illumina sequencing instrument as described in previously published studies from the Greenleaf lab (Buenrostro and Araya et al., Nat. Biotech. 2014).
Data analysis	Custom scripts described in previously published work (Buenrostro and Araya et al., Nat. Biotech. 2014) were used to analyze raw fluorescence images and align them to sequencing data. Further analysis to fit cluster intensity time series to exponential decay models and to fit reaction rates as a function of ligand concentration to a Michaelis-Menten model was performed using standard fitting routines in Python (see Online Methods for specific details).

For manuscripts utilizing custom algorithms or software that are central to the research but not yet described in published literature, software must be made available to editors/reviewers. We strongly encourage code deposition in a community repository (e.g. GitHub). See the Nature Research guidelines for submitting code & software for further information.

Data

Policy information about availability of data

All manuscripts must include a data availability statement. This statement should provide the following information, where applicable:

- Accession codes, unique identifiers, or web links for publicly available datasets
- A list of figures that have associated raw data
- A description of any restrictions on data availability

Most of the data presented in the main text figures, as well as the supplementary figures, have been provided in the Source Data. The Source Data file includes a document which functions as a guide for connecting various source data to their corresponding figures. These source data cover Figs. 1d-e, 2, 3, 4, and 5, and Supplementary Figs. S1c, S2, S3, S4, and S5a. Other data supporting the findings of this study are available from the corresponding authors upon reasonable request.

Field-specific reporting

Please select the one below that is the best fit for your research. If you are not sure, read the appropriate sections before making your selection.

☒ Life sciences ☐ Behavioural & social sciences ☐ Ecological, evolutionary & environmental sciences

For a reference copy of the document with all sections, see nature.com/documents/nr-reporting-summary-flat.pdf

Life sciences study design

All studies must disclose on these points even when the disclosure is negative.

Sample size	No method was used to predetermine sample size. Each variant's cleavage rates were determined from the average behavior of multiple individual RNA clusters (each consisting of ~1000 molecules), e.g., thousands of individual clusters for single mutants, and tens to hundreds of individual clusters for double mutants. This resulted in good reproducibility of the results.
Data exclusions	No data were excluded from the analysis.
Replication	Two independent RNA array measurements of cleavage rates at 10 mM GlcN6P (on two separate sequencing chips) revealed that the measurements were highly reproducible.
Randomization	Randomization was not relevant to this study. Samples were not randomized; the only samples involved were RNA arrays with different ligand concentrations added. Different ligand concentrations gave readily apparent differences in cleavage rates across all variants.
Blinding	Blinding was not relevant to this study. Investigators were not blinded. As noted above, different ligand concentrations gave readily apparent differences in cleavage rates across all variants.

Reporting for specific materials, systems and methods

We require information from authors about some types of materials, experimental systems and methods used in many studies. Here, indicate whether each material, system or method listed is relevant to your study. If you are not sure if a list item applies to your research, read the appropriate section before selecting a response.

Materials & experimental systems

n/a	Involved in the study
☒	☐ Antibodies
☒	☐ Eukaryotic cell lines
☒	☐ Palaeontology
☒	☐ Animals and other organisms
☒	☐ Human research participants
☒	☐ Clinical data

Methods

n/a	Involved in the study
☒	☐ ChIP-seq
☒	☐ Flow cytometry
☒	☐ MRI-based neuroimaging
